# Supplementary material for: Exploring the Relationship between Running-Related Technology Use and Running-Related Injuries: A Cross-Sectional Study of Recreational and Elite Long-Distance Runners
Source: Healthcare (Basel). 2024 Mar 13;12(6):642. doi: 10.3390/healthcare12060642 (PMC10970008; doi:10.3390/healthcare12060642)
Supplement: Supplementary file 1 [file healthcare-12-00642-s001.zip › Figure File S1 Recruitment Flyer.pdf]

WCM IRB
Approved for
use
25-Feb-2021

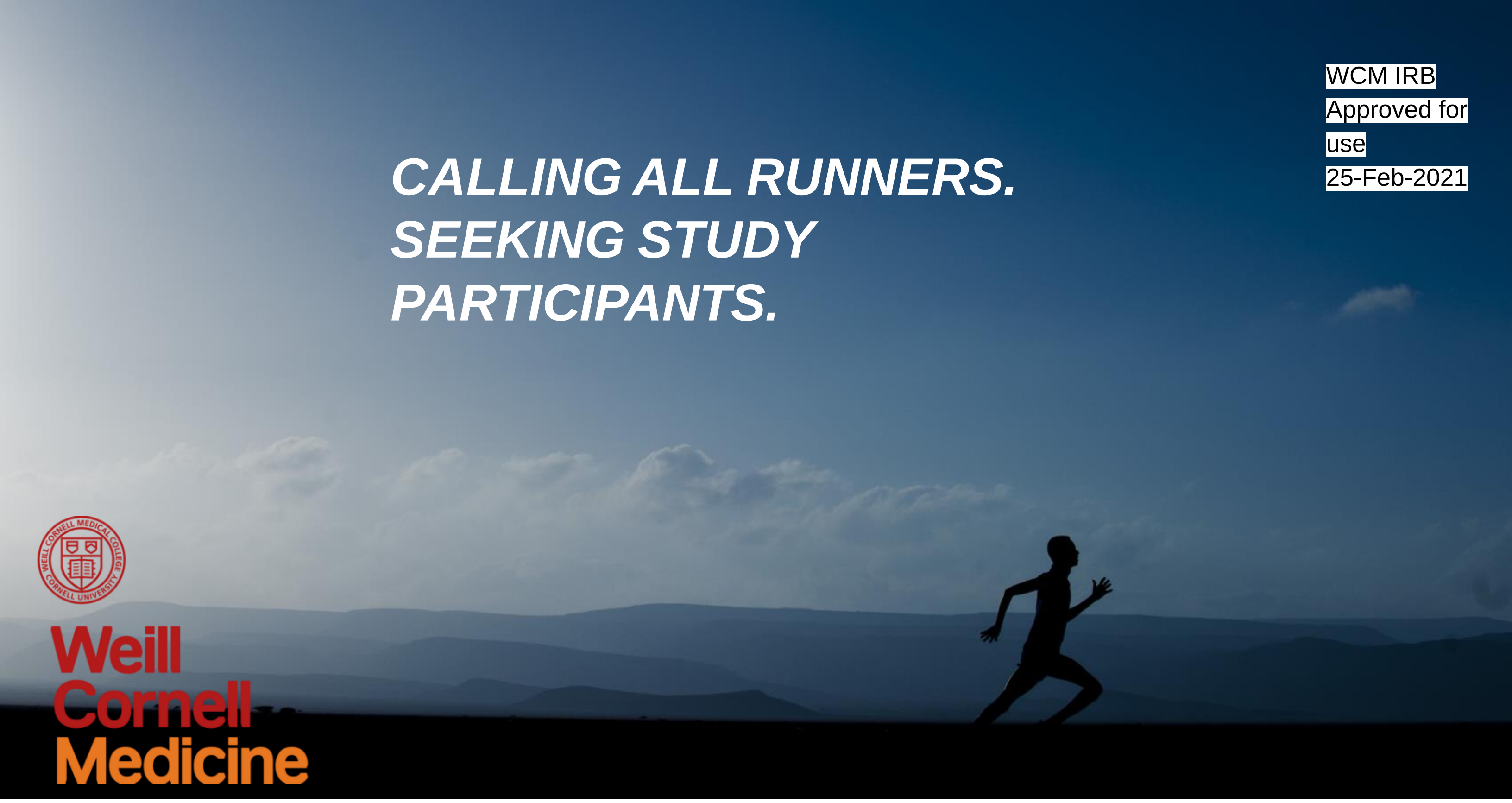

CALLING ALL RUNNERS. SEEKING STUDY PARTICIPANTS.

Weill Cornell Medicine

STUDY AIM?

This study seeks to better understand the current use of wearable technology by runners and the potential relationship with running-related injuries

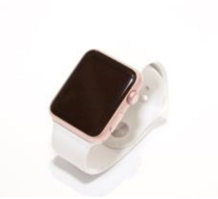

ARE YOU ELIGIBLE?

Currently training or trained & ran in a race ≥10K in the last 12 months

KNOWLEDGE IS POWER

The knowledge gained from this investigation will provide runners with insight into:

- Commonly Tracked Running Metrics
- Latest Running Trends
- Potential Roles of Wearable Technology

QUESTIONS OR ELIGIBILITY INQUIRIES- CONTACT A MEMBER OF OUR STUDY TEAM:

ZACHARY SMITH
ZCS4001@MED.CORNELL.EDU

HOW CAN YOU GET INVOLVED?

You can complete a one-time survey at the following address or by scanning the QR code:

https://redcap.link/wearabletechandrunningsurvey

SCAN ME

Get in touch • zcs4001@med.cornell.edu • https://redcap.link/wearabletechandrunningsurvey
