# Supplementary material for: Exploring the Relationship between Running-Related Technology Use and Running-Related Injuries: A Cross-Sectional Study of Recreational and Elite Long-Distance Runners
Source: Healthcare (Basel). 2024 Mar 13;12(6):642. doi: 10.3390/healthcare12060642 (PMC10970008; doi:10.3390/healthcare12060642)
Supplement: Supplementary file 1 [file healthcare-12-00642-s001.zip › Supplementary file S2 Sample Survey.pdf]

Wearable Technology and Running Survey

This survey is for research purposes only. The purpose of this survey is to collect data on wearable technology usage in runners. Please only fill out if you have trained for a race ≥10 km in the past 12 months. No identifying information will be collected and all participation is voluntary. By completing this survey, you are agreeing to have your answers included in this research investigation.

1. **Age:** _______________

2. **Sex:**

- ○ Female
- ○ Male
- ○ Other: ____________________

3. **Ethnicity:**

- ○ Asian/Pacific Islander
- ○ Black/African American
- ○ Hispanic/Latino
- ○ Native American
- ○ White
- ○ Other: ____________________

4. **How Long Have You Been Running regularly?**

- ○ 0-5 Years
- ○ 6-10 Years
- ○ 11-15 Years
- ○ 16-20 Years
- ○ 21+ Years

5. **Which of the Following Best Describes You?**

- ○ Run Recreationally to Stay Fit – Do Not Participate in Organized Competition
- ○ Run 1-3 Races Per Year
- ○ Run 4-6 Races Per Year
- ○ Run 7-9 Races Per Year
- ○ Run 10+ Races Per Year
- ○ Run at the Elite Level (Collegiate or Professional)

6. **What Events Do You Typically Train For (click all that apply)?**

- ○ 10 km (6.2 miles)
- ○ 15 km (9.3 miles)
- ○ 20 km (12.4 miles)
- ○ Half-Marathon (13.1 miles)
- ○ 25 km (15.5 miles)
- ○ 30 km (18.6 miles)
- ○ Marathon (26.2 miles)

○ Ironman ○ N/A ○ Other: _______ _____________

○ Ultramarathon

7. In the Past 12 months, How Many Events Did You Train For and Run?

---

8. Are You Currently Training for An Event?

○ Yes

○ No

9. How Many Days Per Week Do You Typically Run?

○ 0-1 Days/Week ○ 4-5 Days/Week

○ 2-3 Days/Week ○ 6-7 Days/Week

10. How Many Miles Per Week Do You Typically Run?

○ 0-10 Miles

○ 11-20 Miles

○ 21-30 miles

○ 31-40 Miles

○ 40+ Miles

11. Do You Ever Use Smart Technology to Track runs? i.e. Smart Watch or Phone Application?

○ Yes

○ No

○ Sometimes

12. If Yes To The Question Above, Do You Use a Smartwatch Or An Application On Your Phone to Track Data?

○ Smartwatch

○ Smartphone App

○ Both

○ Other: ___________________

13. If You Use A Smartwatch, Which Device Brand Are You Currently Using?

○ Apple Watch
○ Garmin
○ Fitbit
○ Polar
○ Whoop
○ Timex
○ Other: ___________________

14. What Parameters Do You Typically Track (click all that apply)?

○ Activity Time
○ Heart Rate
○ Heart Rate Zone
○ Respiratory Rate
○ VO2max
○ Distance
○ Pace
○ Calories
○ Altitude Change
○ Cadence
○ Stride Length
○ Ground Contact Time
○ Distance Improvement
○ Pace Improvement
○ Other: ______ ____________

15. Do You Use This Information To Make Future Training Decisions?
i.e. Answering yes to this question means that the information provided in the previous question is used to adjust for training variables such as distance, pace, or running frequency in the proceeding days

○ Yes
○ No
○ Sometimes

16. While Training For A competitive Event ≥10 km Race In The Last Year, Did You Suffer A running-Related Injury?

A Running-Related Injury (RRI) is defined in this study as an injury sustained from running that caused a modification in distance, pace, or frequency OR caused you to stop running for 7 days or 3 consecutive scheduled training sessions.

○ Yes
○ No

17. Approximately how many running related injuries did you sustain while training for your event? ________

18. What Parts of the Body Were Affected By Injury? (Select all that apply)

- ○ Hip
- ○ Knee
- ○ Ankle
- ○ Foot
- ○ Achilles Tendon
- ○ Lower Leg
- ○ Hamstring
- ○ Lower Back
- ○ Other: ___________________

19. Have You Injured That Part of The Body Previously?

- ○ Yes
- ○ No

20. When Returning Back To Running After An Injury, What Metrics on your smart device Do You Use To Guide Your Training?

- ○ Activity Time
- ○ Heart Rate
- ○ Heart Rate Zone
- ○ Respiratory Rate
- ○ Distance
- ○ Pace
- ○ Calories
- ○ Altitude Change
- ○ Cadence
- ○ Stride Length
- ○ Ground Contact Time
- ○ Distance Improvement
- ○ Pace Improvement
- ○ Other: _______ _____________

21. Do You Feel That Using Wearable Technology Helps To Prevent Running-Related Injuries?

- ○ Yes
- ○ No

22. In The Last 12 months, Did You Receive a Positive PCR test for COVID-19?

- ○ Yes
- ○ No

23. Did You Track any Metrics During This Period With Your Smart device?

- ○ Yes
- ○ No

24. If So, Which Metrics Did You Track?

- ○ Activity Time
- ○ Heart Rate
- ○ Heart Rate Zone
- ○ Respiratory Rate
- ○ Distance
- ○ Pace
- ○ Calories
- ○ Altitude Change
- ○ Cadence
- ○ Stride Length
- ○ Ground Contact Time
- ○ Distance Improvement
- ○ Pace Improvement
- ○ Other: ____________________

25. Did You Notice Any Changes in These Metrics?

- ○ Yes
- ○ No

26. If So, Which Metrics Did You Notice A Change In?

- ○ Activity Time
- ○ Heart Rate
- ○ Heart Rate Zone
- ○ Respiratory Rate
- ○ Distance
- ○ Pace
- ○ Calories
- ○ Altitude Change
- ○ Cadence
- ○ Stride Length
- ○ Ground Contact Time
- ○ Distance Improvement
- ○ Pace Improvement
- ○ Other: ____________________

27. How long after diagnosis of COVID19 did you resume starting to run? __________

28. Did you notice any difference in your metrics on your smart watch when resuming running again?

- o **Yes**
- o **No**

29. If So, Which Metrics Did You Notice A Change In?

- ○ Activity Time
- ○ Heart Rate
- ○ Heart Rate Zone
- ○ Respiratory Rate

- Distance
- Pace
- Calories
- Altitude Change
- Cadence
- Stride Length
- Ground Contact Time

30. Do You Feel That Smartwatches Provide an Accurate Means for Tracking Non-Exercise Metrics?

- Yes
- No
